# Supplementary material for: Sleeping Beauty Transposon Insertions into Nucleolar DNA by an Engineered Transposase Localized in the Nucleolus
Source: Int J Mol Sci. 2023 Oct 7;24(19):14978. doi: 10.3390/ijms241914978 (PMC10573994; doi:10.3390/ijms241914978)
Supplement: Supplementary file 1 [file ijms-24-14978-s001.zip › Figure S1.pdf]

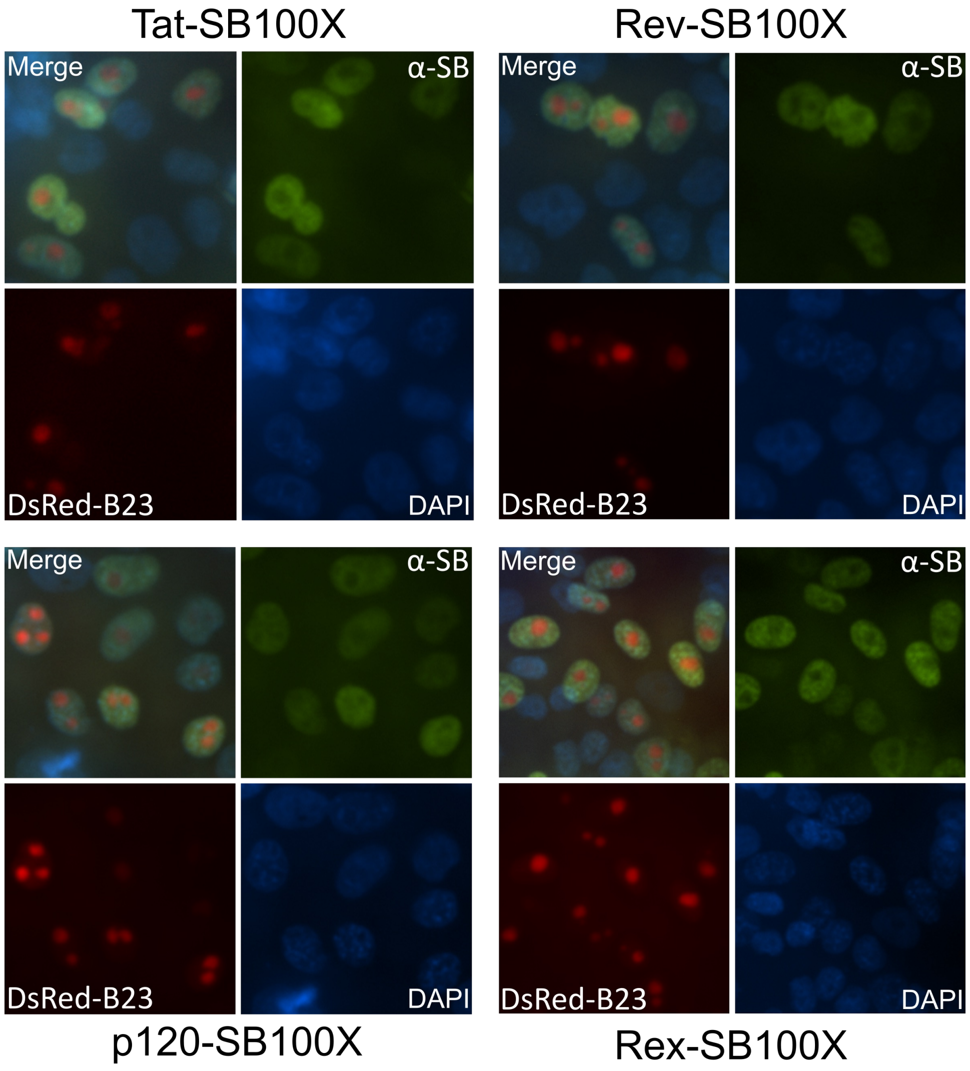

Supplementary Figure S1. Subnuclear distribution of the *Sleeping Beauty* transposase fused to four short NoLSs. Subcellular localization of the *Sleeping Beauty* transposase was highlighted with a transposase-specific antibody (green), while B23 fused to a red fluorescent protein (DsRed-B23) was used as a nucleolar marker.
