# Supplementary material for: Sleeping Beauty Transposon Insertions into Nucleolar DNA by an Engineered Transposase Localized in the Nucleolus
Source: Int J Mol Sci. 2023 Oct 7;24(19):14978. doi: 10.3390/ijms241914978 (PMC10573994; doi:10.3390/ijms241914978)
Supplement: Supplementary file 1 [file ijms-24-14978-s001.zip › Figure S2.pdf]

A

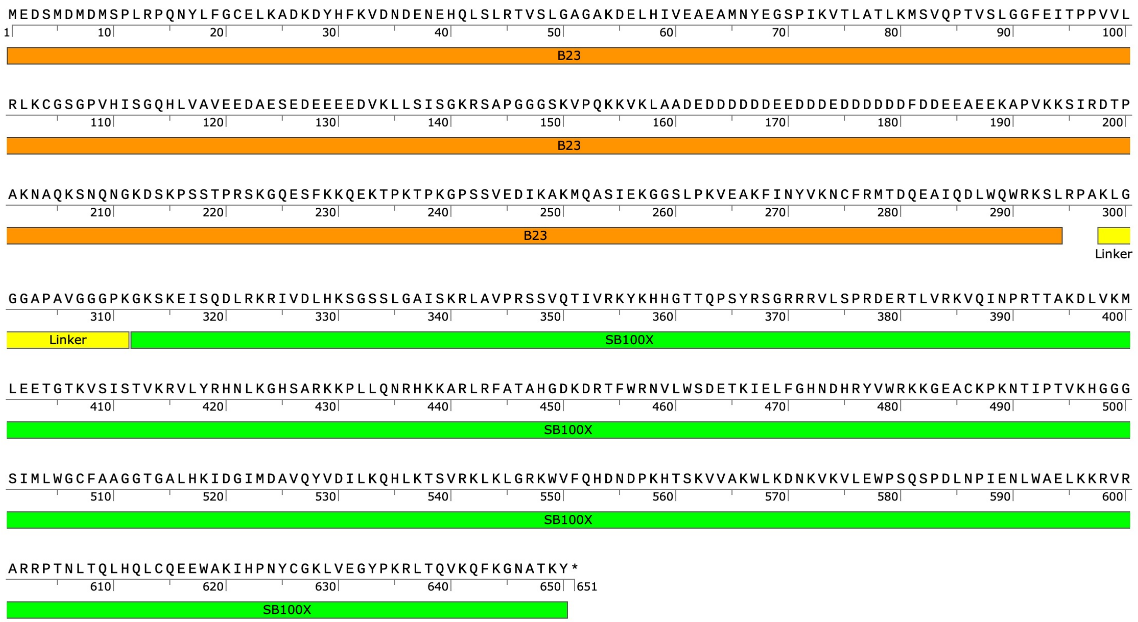

B

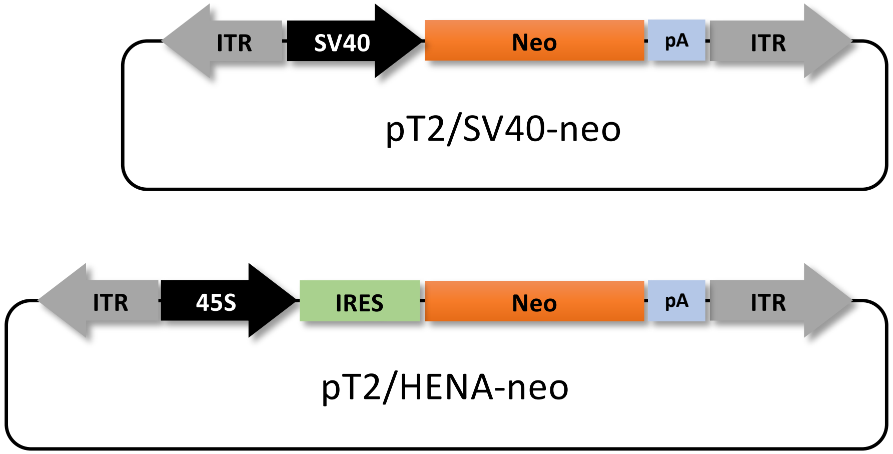

Supplementary Figure S2. The major components driving *Sleeping Beauty* transposon insertions in this study. (A) Amino acid sequence of the B23-SB100X fusion protein. The B23 protein (orange) is fused to the SB100X transposase (green) through a flexible linker (yellow). **(B)** Overall architecture and functional elements of the two transposon vectors used in this study. The Sleeping Beauty transposon elements are maintained as plasmids, the transgene cassettes including the SV40 or 45S Pol I promoters, an internal ribosome entry sequence (IRES), a selectable neomycin resistance gene (Neo) and a polyadenylation site (pA) are flanked by the inverted terminal repeats (ITR) of the *Sleeping Beauty* transposon.
