# Supplementary material for: Sleeping Beauty Transposon Insertions into Nucleolar DNA by an Engineered Transposase Localized in the Nucleolus
Source: Int J Mol Sci. 2023 Oct 7;24(19):14978. doi: 10.3390/ijms241914978 (PMC10573994; doi:10.3390/ijms241914978)
Supplement: Supplementary file 1 [file ijms-24-14978-s001.zip › Figure S3.pdf]

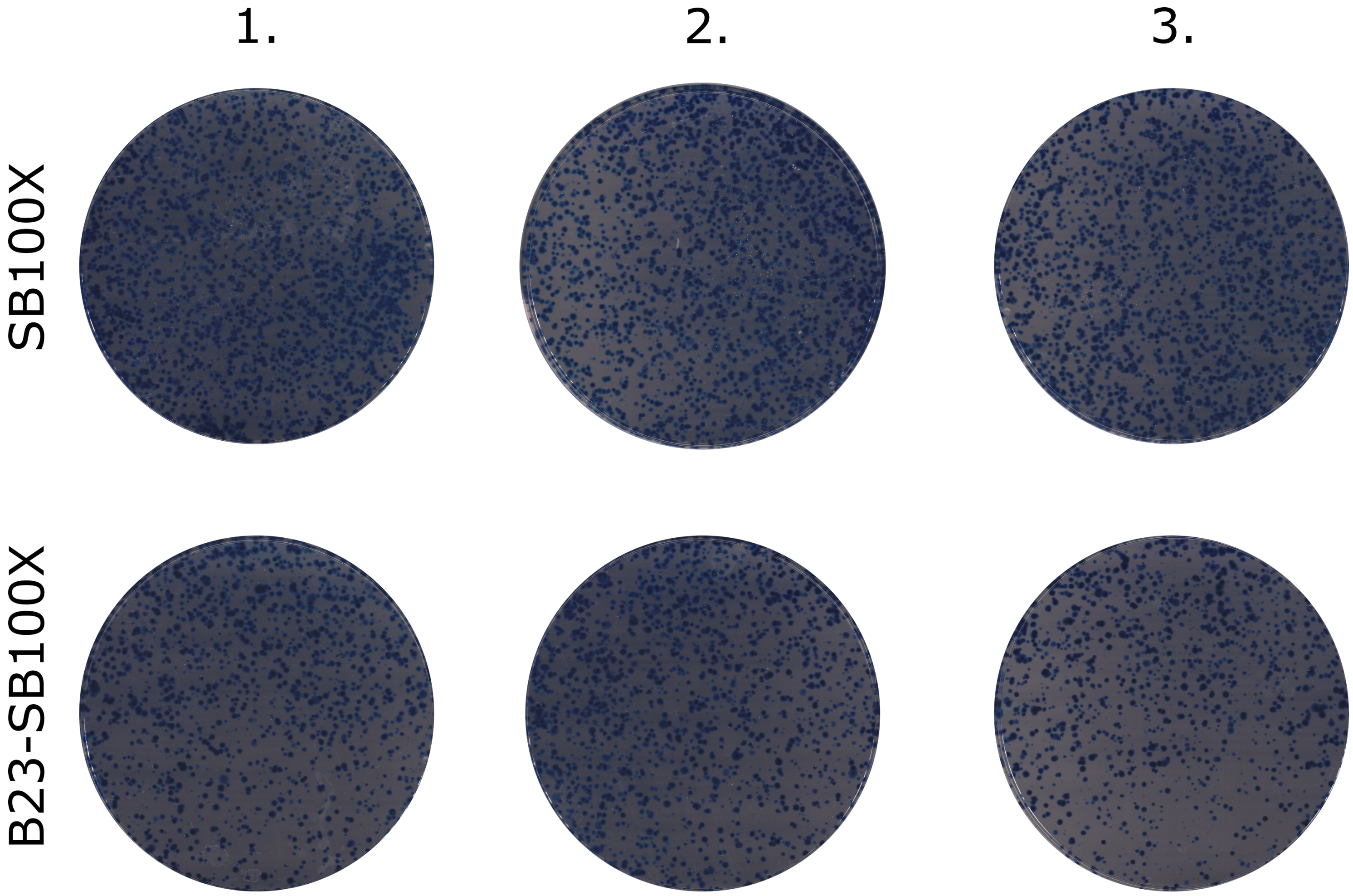

Supplementary Figure S3. Antibiotic-resistant cell colonies generated in the transposition assays described in Figure 2B. Plates of an experiment executed in triplicates were stained using methylene blue for visualization.
