# Supplementary material for: Sleeping Beauty Transposon Insertions into Nucleolar DNA by an Engineered Transposase Localized in the Nucleolus
Source: Int J Mol Sci. 2023 Oct 7;24(19):14978. doi: 10.3390/ijms241914978 (PMC10573994; doi:10.3390/ijms241914978)
Supplement: Supplementary file 1 [file ijms-24-14978-s001.zip › Figure S4.pdf]

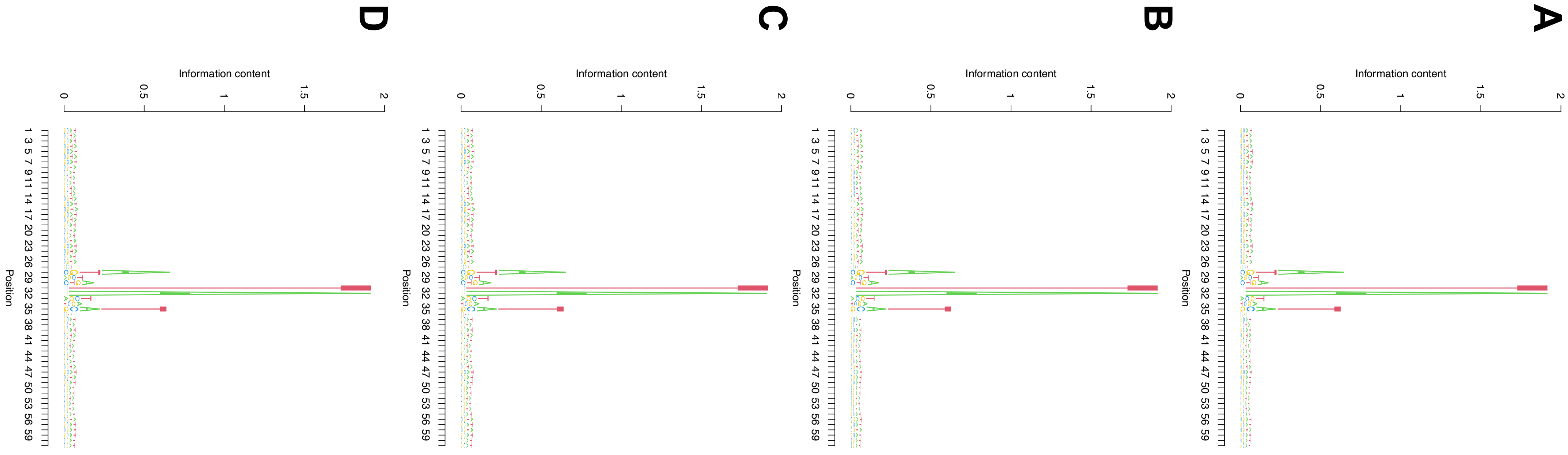

Supplementary Figure S4. Sequence analysis of transposon integration sites. The sequence logos show the base frequencies of nucleotides at the genomic insertion loci in a 60-bp window, centered around the target TA dinucleotides. The relative size of the letters in each position of the logo corresponds to the frequency of the bases in the integration loci. The value 2 ($log_2 4$) on the *y* axis stands for maximum possible frequency. We harnessed the SeqLogo analysis also for the quality control of the 'random' vs 'unique' genome alignment settings (detailed in Materials and Methods) The A-B and C-D panels show the SeqLogos of the B23HENA and B23SV conditions obtained with the 'random' (A, C) or with the 'unique' (B, D) settings, respectively. The high similarity of the logos obtained with the two mapping strategies suggest that the two methods identified insertion sites with similar fidelity.
