# Supplementary material for: Sleeping Beauty Transposon Insertions into Nucleolar DNA by an Engineered Transposase Localized in the Nucleolus
Source: Int J Mol Sci. 2023 Oct 7;24(19):14978. doi: 10.3390/ijms241914978 (PMC10573994; doi:10.3390/ijms241914978)
Supplement: Supplementary file 1 [file ijms-24-14978-s001.zip › Figure S5.pdf]

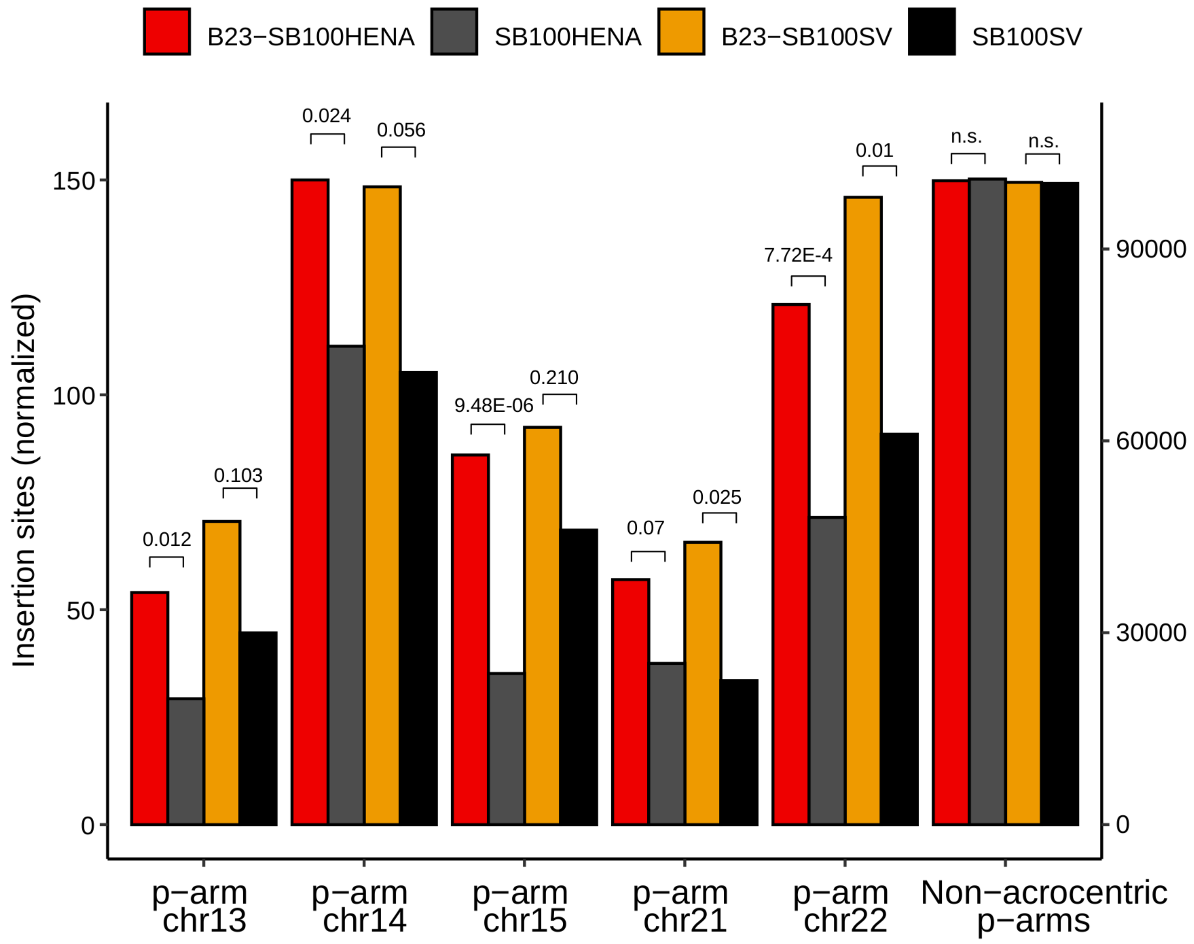

Supplementary Figure S5. Frequency of insertions on the *p*-arms of each chromosome of the nucleolus. The second *y*-axis applies to the insertion sites on the non-acrocentric chromosomes. The P values above the bars are of the Fisher's exact test.
