# Supplementary material for: Sleeping Beauty Transposon Insertions into Nucleolar DNA by an Engineered Transposase Localized in the Nucleolus
Source: Int J Mol Sci. 2023 Oct 7;24(19):14978. doi: 10.3390/ijms241914978 (PMC10573994; doi:10.3390/ijms241914978)
Supplement: Supplementary file 1 [file ijms-24-14978-s001.zip › Figure S6.pdf]

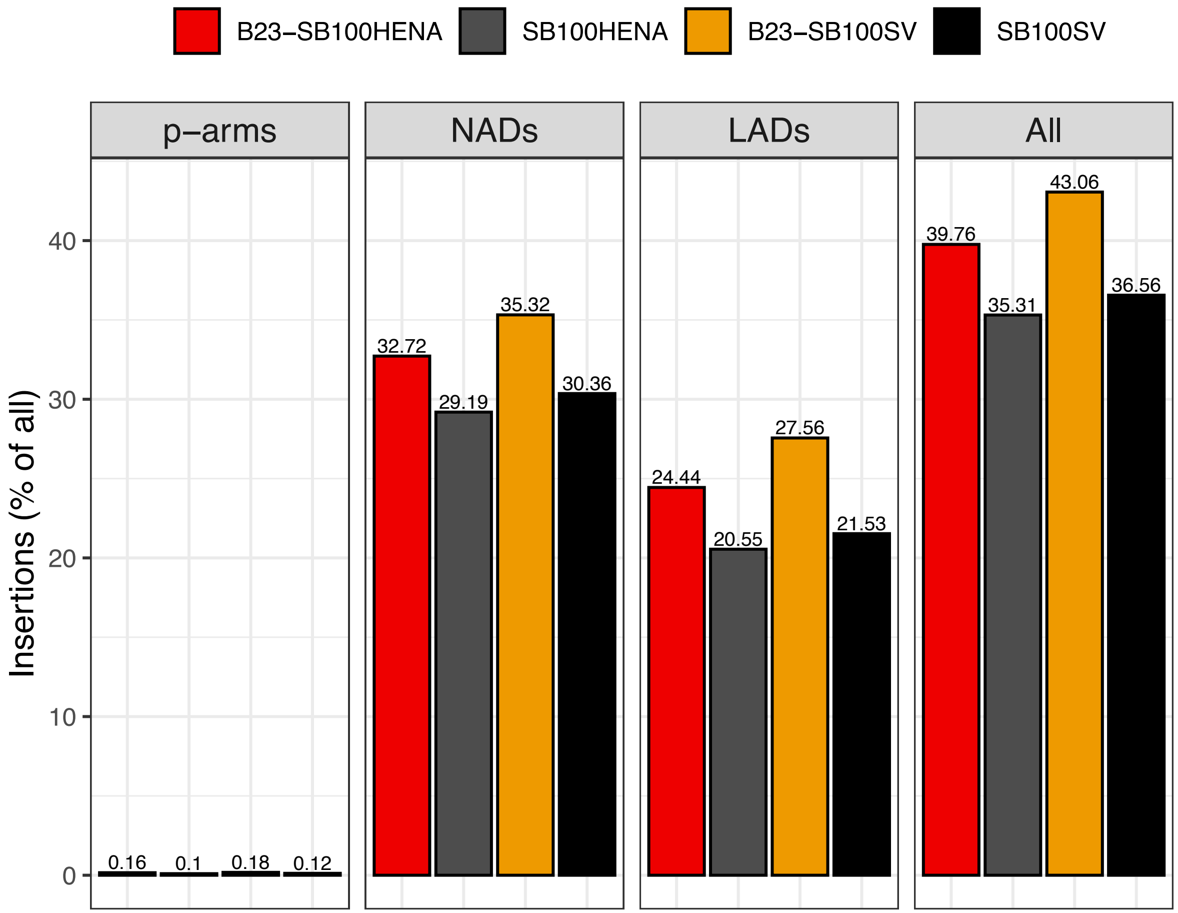

Supplementary Figure S6. Frequency of transposon insertions within and in the vicinity of the nucleolar DNA. The percentage of all insertions per conditions are shown on the *y*-axis. The genomic regions of interest are indicated above. The nucleolar DNA, in its strictest sense, is represented by *p*-arms of the NOR-containing acrocentric chromosomes (p-arms). Nucleolus-associated chromatin domains (NADs) and lamina-associated domains (LADs) are the chromatin regions, which are known to be present in the proximity of the nucleolar DNA. The figure shows that only a small fraction of the total insertions mapped near to the arrays of rRNA genes, and that the majority of the insertion events took place in the nucleolus-associated DNA. The category “All” stands for the unified regions of the previous three. Of note, the NAD and the LAD categories contain chromosomal segments which often overlap.
