# Supplementary material for: Sleeping Beauty Transposon Insertions into Nucleolar DNA by an Engineered Transposase Localized in the Nucleolus
Source: Int J Mol Sci. 2023 Oct 7;24(19):14978. doi: 10.3390/ijms241914978 (PMC10573994; doi:10.3390/ijms241914978)
Supplement: Supplementary file 1 [file ijms-24-14978-s001.zip › Figure S7.pdf]

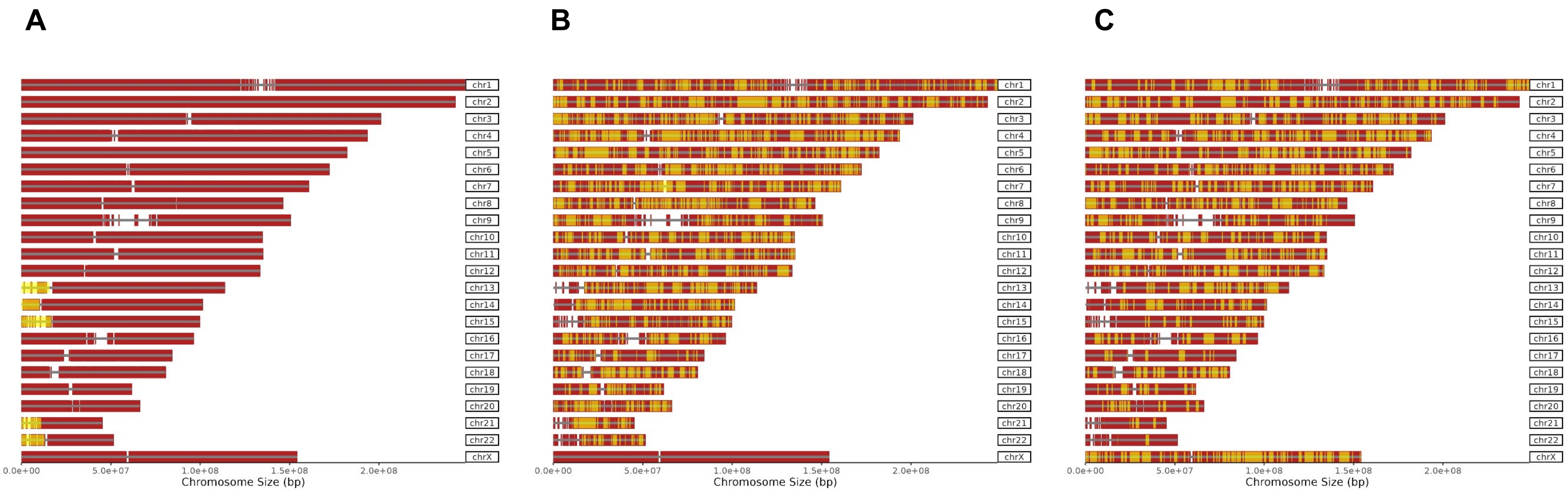

Supplementary Figure S7. Distribution of transposon insertion sites on the human chromosomes. The chromosome names are listed on the right. The thin gray lines represent the entire lengths of each chromosome. Insertion loci are marked in red. The yellow segments correspond to regions of interest: (**A**) *p*-arms of the NOR-containing acrocentric chromosomes, (**B**) nucleolus-associated chromatin domains (NADs), (**C**) lamina-associated domains (LADs). The figure depicts an overall genome-wide distribution of the integrations on the chromosomes. Centromeric and telomeric regions (including the NORs) tend to be less densely populated by insertions, due either to chromatin factors and/or to the repetitive nature of these regions.
