# Supplementary material for: Sleeping Beauty Transposon Insertions into Nucleolar DNA by an Engineered Transposase Localized in the Nucleolus
Source: Int J Mol Sci. 2023 Oct 7;24(19):14978. doi: 10.3390/ijms241914978 (PMC10573994; doi:10.3390/ijms241914978)
Supplement: Supplementary file 1 [file ijms-24-14978-s001.zip › Supplementary Table S1.pdf]

Supplementary Table S1. Oligonucleotide sequences

Overhangs (non-binding regions) are underlined, restriction sites are **bold**, filler sequences are in lowercase, and * marks a phosphorothioate bond.

The primers PE_nest_BC and SB20hmr_BC contain hexanucleotide barcodes (represented by NNNNNN in the sequence).

**Primer name**	**Sequence (5’→3’)**
**Primers for expression constructs**	
Tat-SB100X_fwd	AGAGAAGAAGAGCCCACCAGAACGGAAAATCAAAAGAAATCAGCCAAGACCTC
Tat-SB100X_rev	GTCTTCTCTTCTTTCTGCCCATGGTACTAGTCCCTATAGTGAGTCGTAT
Rev-SB100X_fwd	AGAAGAAGATGGAGAGAGAGACAGAGACAGGGAAAATCAAAAGAAATCAGCCAAGACC
Rev-SB100X_rev	TCTGTTTCTTCTGGCCTGTCTGCCCATGGTACTAGTCCCTATAGTGAGTCGTAT
p120-SB100X_fwd	AGAAAGAGAGCCGCCAAGAGAAGACTGGGAAAATCAAAAGAAATCAGCCAAGACCTC
p120-SB100X_rev	GGCTCTGCTGCTCAGTCTCTTGCTGCCCATGGTACTAGTCCCTATAGTGAGTCGTAT
Rex-SB100X_fwd	AGAAGCCAGAGAAAGAGACCTCCCACCCCTGGAAAATCAAAAGAAATCAGCCAAGACCTC
Rex-SB100X_rev	TCTGGGTCTTCTTCTGGTCTTGGGGCCCATGGTACTAGTCCCTATAGTGAGTCGTAT
L-SB100X_fwd1	ACCTGCTGTGGGCGGAGGCCCTAAGATGGGAAAATCAAAAGAAATCAGCCAAGAC

FseI-L-SB100X_fwd2	aatc**GGCCGGCC**AAACTGGGCGGAGGCGCACCTGCTGTGGGCGGAG
NotI-SBT7_rev	aatc**GCGGCCGC**AGTCCCTATAGTGAGTCGTATTAATTCCTTCCG
NotI-S-B23_fwd	aatc**GCGGCCGC**ATGTATGGAAGATTCGATGGACATGGACATGA
FseI-B23_rev	aatc**GGCCGGCC**tAAGAGACTTCCTCCACTGCCAGA

Other primers

PE_nest_BC	CAAGCAGAAGACGGCATACGAGATNNNNNNGTGACTGGAGTTCAG
SB20hmr_BC	AATGATACGGCGACCACCGAGATCTACACTCTTTCCCTACACGACGCTCTTCCGATCTNNNNNNACTTAAGTGTATGTAAACTTCCGACT
T-bal_long	CTTGTGTCATGCACAAAGTAGATGTCCTAACTGACT
TS_linker	GTAATACGACTCACTATAGGGC
Linker_TruSeq_T+	GTAATACGACTCACTATAGGGCTCCGCTTAAGGGACTCAGACGTGTGCTCTTCCGATC*T
Linker_TruSeq_T-	GATCGGAAGAGCACACG-SPACERC3
